# Clinical utility of diffusion tensor imaging in sport-related concussion: a systematic review

**DOI:** 10.1093/bjro/tzaf024

**Published:** 2025-10-08

**Authors:** Shiv Patil, Rithvik Kata, Serhat Aydin, Mert Karabacak, Konstantinos Margetis, Sotirios Bisdas

**Affiliations:** Sidney Kimmel Medical College, Thomas Jefferson University, Philadelphia, PA 19107, United States; Sidney Kimmel Medical College, Thomas Jefferson University, Philadelphia, PA 19107, United States; School of Medicine, Koç University, Istanbul, 34450, Turkey; Department of Neurosurgery, Mount Sinai Health System, New York, NY 10029, United States; Department of Neurosurgery, Mount Sinai Health System, New York, NY 10029, United States; Department of Neuroradiology, University College London Hospitals NHS Foundation Trust, London WC1N 3BG, United Kingdom; Department of Brain Repair and Rehabilitation, Queen Square Institute of Neurology, University College London, London, WC1N 3BG, United Kingdom

**Keywords:** diffusion tensor imaging, sport-related concussion, MRI, white matter integrity

## Abstract

**Objective:**

Sport-related concussion (SRC) is a prevalent form of traumatic brain injury that is associated with long-term neurological and psychiatric impairment, particularly among athletes with a history of repetitive concussions. The biological variability of SRC’s impact on the brain, as well as a lack of objective biomarkers to diagnose and prognosticate concussion, has prompted interest in advanced neuroimaging methods such as diffusion tensor imaging (DTI). By measuring disruptions in water diffusivity due to head trauma, DTI can detect alterations in white matter integrity that are not visualized by conventional imaging methods. This systematic review aims to synthesize major trends and findings on original research studies that utilized DTI to evaluate subjects for SRC.

**Methods:**

An initial search from PubMed, Web of Science, and Scopus generated 397 articles published from database inception to 2024, with 26 studies included in the final qualitative synthesis.

**Results:**

Findings showed heterogenous changes in DTI parameters during acute injury with more consistent alterations seen in chronic injury, particularly as reduced fractional anisotropy and elevated mean diffusivity. Significant variability was observed in study design and methodology, which may explain discrepancies in findings across studies.

**Conclusions:**

Future research efforts should implement standardized methods capable of accounting for inter-individual differences to further validate DTI’s role as an objective biomarker of SRC.

**Advances in knowledge:**

Individualized analysis of DTI could serve as a diagnostic tool and prognostic metric for patients with SRC, thus enabling an objective measure of long-term outcome and suitability for return-to-play.

## Introduction

Sport-related concussion (SRC) is a form of traumatic brain injury (TBI) induced by biomechanical forces that lead to transient disturbances in brain function.^1^ Comprising one-third of all TBIs, SRC is estimated to affect 2%-15% of athletes participating in organized sports each season, with a disproportionate incidence observed among adolescents and young adults.^2^^,^^3^ The rising incidence of SRC, coupled with growing evidence linking head trauma to long-term cognitive impairment, has positioned the accurate evaluation of SRC and its sequelae as a critical public health priority.^4^^,^^5^

The diagnosis of SRC remains a significant challenge due to the lack of standardized, objective criteria and continues to rely heavily on clinical judgment.^6^ The Sports Cognitive Assessment Tool 6 (SCAT6) is one of the most widely used methods to evaluate SRC, beginning with an on-field assessment of red flag signs that necessitate removal from play and immediate medical intervention.^7^ The SCAT6 subsequently combines various clinical scales to examine symptoms and impairment of cognitive or motor function. While this approach has introduced a degree of homogeneity to the diagnostic workup of SRC, concerns have persisted surrounding the reliability of its criteria.^8^

Neuroimaging is typically reserved for cases of SRC where there is high clinical suspicion for a neurosurgical emergency, such as intracranial bleeding or skull fracture.^2^ In the acute setting, non-contrast CT is preferred, with MRI considered at follow-up for cases with chronic symptoms.^9^ While these conventional imaging techniques can detect structural alterations associated with SRC, their diagnostic and prognostic value remains limited.^10^ This limitation may be attributed to the presence of subtle microstructural changes in the brain following head trauma, such as diffuse axonal injury, which often lie below the detection threshold of conventional CT or MRI.^11^

Novel developments in neuroimaging provide promising methods for identifying clinically relevant alterations in the brain associated with SRC and recovery.^12^ Susceptibility weighted imaging is an advanced MRI sequence highly sensitive to traumatic microbleeds and hemorrhagic lesions that may correlate with injury severity and long-term cognitive outcome.^13^ Diffusion tensor imaging (DTI) is another MRI modality capable of visualizing microstructural changes in white and gray matter tracts by measuring patterns of water diffusion, a process that is naturally restricted by the architecture of myelinated axons.^14^ Quantitative measures that can be obtained from DTI include fractional anisotropy (FA, the directionality of water diffusion), mean diffusivity (MD, the molecular diffusion rate), axial diffusivity (AD, the diffusion rate along the parallel axis), and radial diffusivity (RD, the diffusion rate along the perpendicular axis). Table 1 provides further details of these DTI parameters and their relevance to quantifying white matter (WM) integrity.

**Table 1.** Description of conventional measures obtained from DTI and their relevance to white matter integrity.

DTI measure	Description
Fractional anisotropy (FA)	A scalar value (0-1) that reflects the directionality (anisotropy) of water diffusion in tissue. Low FA may indicate demyelination or a loss of coherence in the preferred diffusion direction as a result of axonal injury.
Mean diffusivity (MD) (also known as apparent diffusion coefficient [ADC])	The average rate of water diffusion across all directions (trace of the diffusion tensor). High MD suggests increased free diffusion in damaged tissue.
Radial diffusivity (RD)	The rate of diffusion perpendicular to the principal axis of a white matter fiber. Alterations in RD are consistent with white matter demyelination.
Axial diffusivity (AD)	The rate of water diffusion parallel to the main axis of a white matter fiber. Compared to RD, AD is more specific to axonal degeneration. Demyelination has little influence.

Abbreviations: ADC = apparent diffusion coefficient; AD = axial diffusivity; FA = fractional anisotropy; MD = mean diffusivity; RD = radial diffusivity.

Research applying DTI to TBI has demonstrated alterations in diffusivity measures associated with head trauma, as injury-induced axonal swelling and degeneration influence the directionality of water.^15^ However, the clinical relevance of these findings remains relatively unknown, given the variability in study design and parameters used to assess patient status and recovery. The aim of this systematic review is to evaluate original research studies that applied DTI to assess microstructural injury in patients with SRC, focusing on study quality, trends in findings, and clinical correlates.

## Methods

### Search strategy, study selection, and data extraction

The initial search was conducted using PubMed, Web of Science, and Scopus databases from their inception to the date of the final search on 20 October 2024. A thorough search was performed with these databases to ensure feasibility and avoid duplication, as they provide broad and comprehensive coverage of the biomedical literature relevant to SRC and neuroimaging. The following keywords and word combinations were used to specifically capture relevant literature on SRC: “concussion,” “diffusion tensor imaging,” “sport,” “athlete,” and “diagnosis” (Supplementary File S1). This review was conducted in a manner consistent with the guidelines of the Preferred Reporting Items for Systematic Review and Meta-Analyses (PRISMA) (Supplementary File S2).^16^ Overall, ethical approval or informed consent status was explicitly reported across all included studies.

Articles warranted inclusion in this review if they (1) were conducted on human participants evaluated for SRC, (2) used DTI to assess microstructural alterations in concussed athletes, (3) reported statistical analysis results of the relationship between clinical symptoms of SRC and DTI measures, and (4) were original research studies published in a peer-reviewed scientific journal.

Articles were excluded from this review if they (1) were conducted on animals; (2) were not original research studies, ie, systematic reviews, conference abstracts, meta-analyses; (3) were case reports or case series with <5 subjects; (4) were further publications from the same cohort unless the DTI processing methodology in the subsequent studies was different; (5) were not published in English; (6) included patients with TBI from causes unrelated to sports; (7) only performed methodological comparisons, such as correlations with functional imaging or blood-based biomarkers.

All retrieved articles were then imported into Covidence, and any duplicates were removed. Two independent reviewers (R.K. and S.A.) performed an initial title and abstract screen of each study based on the inclusion/exclusion criteria. Conflicts were resolved through the intervention of a third reviewer (S.P.). Full-text articles were then screened for eligibility, with the following data extracted if they were included in the final analysis: first author and year of publication, study country, total number of study participants, number of patients in subgroups (SRC and controls), mean age of participants, interval from injury to scan, type of sport, level of play, DTI quantification method, reported effects on DTI metrics, and relevant clinical outcome measures of SRC symptom(s).

### Quality assessment

Due to substantial variability among published studies in defining and reporting outcome measures for SRC, a qualitative synthesis was performed to highlight key trends and evaluate credibility. Quality Assessment of Diagnostic Studies 2 (QUADAS-2) protocol was performed by 2 reviewers (R.K. and S.A.) to assess the risk of bias for each included study.^17^ The criteria for judgement and responses to signaling questions in the QUADAS-2 protocol are detailed in Supplementary File S3.^10^^,^^18^ Conflicts were resolved through the intervention of a third reviewer (S.P.).

## Results

### Study selection

**Figure 1.**
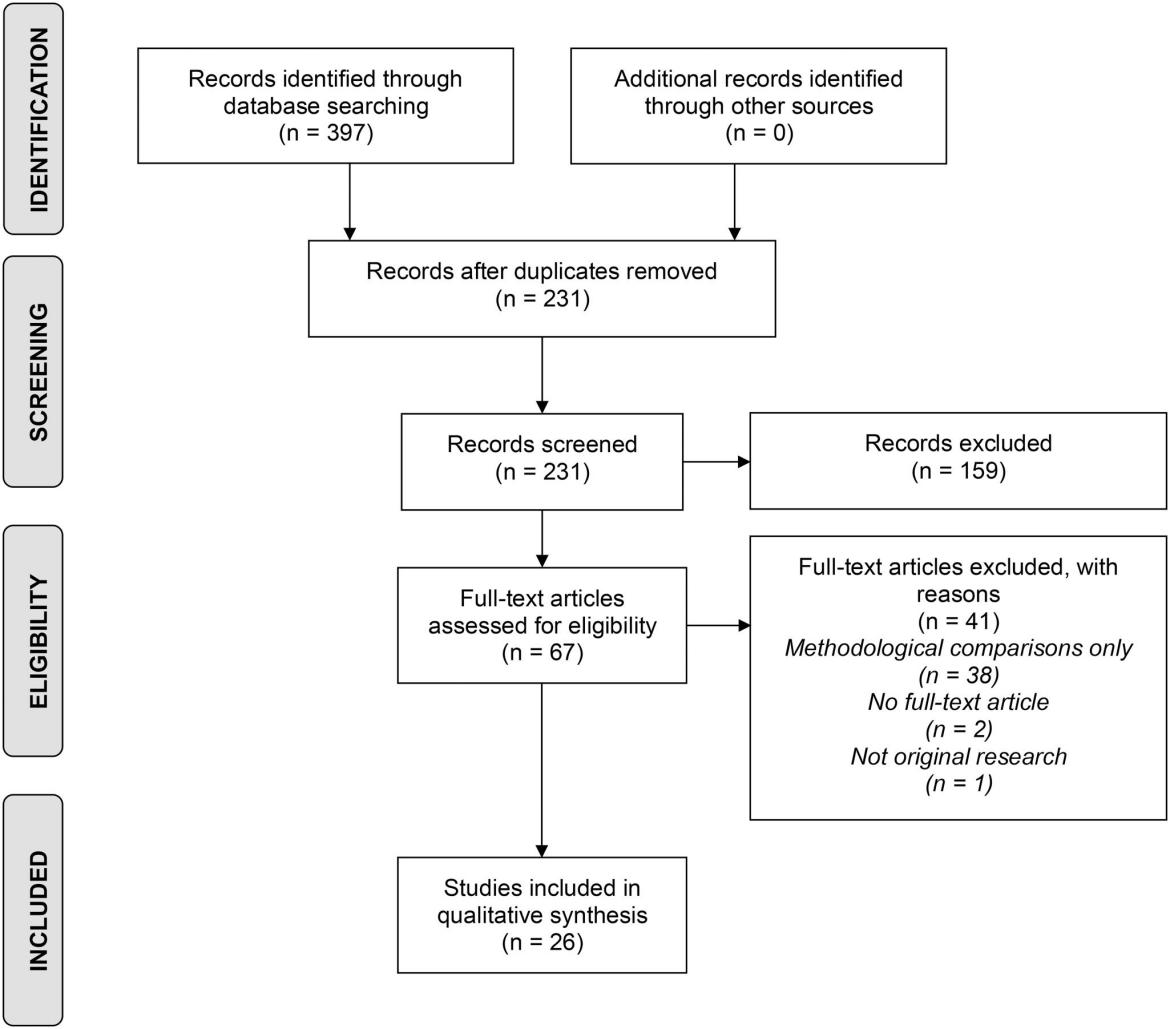
PRISMA diagram of the inclusion and exclusion of studies.

A total of 397 studies were identified using the search strategy. After the removal of 166 duplicates, 231 studies underwent title and abstract screening. Sixty-seven full-text studies were then screened for eligibility based on inclusion and exclusion criteria. An additional 41 studies were removed during this step, resulting in 26 studies that were included in the final qualitative analysis. An overview of the search process is detailed in a PRISMA diagram (Figure 1).

### Study characteristics

Tables 2 and 3 summarize the characteristics of the 26 original studies. The total study population ranged from 15 to 219. The number of patients evaluated for SRC ranged from 10 to 98, while the number of controls ranged from 5 to 137. Fourteen studies only assessed male subjects. Seven studies had a roughly even ratio of male and female subjects (40%-60% males), and 5 had a greater proportion of males. The type of sport differed across studies: football (9/26 studies), ice hockey (2/26), boxing (2/26), Australian football (2/26), soccer (1/26), and a variety of sports (10/26) were represented. Level of play ranged from youth recreational leagues to the professional level.

**Table 2.** Summary of study design and characteristics for the included studies.

First author	Year	Country	Total number of study participants	Number of participants with SRC	Mean age of SRC group, years (SD)	Number of controls	Mean age of control group, years (SD)	Time from injury to scan (SD)
Zhang	2006	USA	68	49 (49 M)	30 (5)	19 (19 M)	3332 (10)	NR
Cubon	2011	Canada	20	10 (5 M)	20 (2)	10 (5 M)	20 (2)	115 (104 d)
Hart	2013	USA	60	34 (34 M)	62	26 (26 M)	60	NR
Strain	2013	USA	48	26 (26 M)	58 (12)	22 (22 M)	59 (12)	9 (4 y)
Bazarian	2014	USA	15	10 (10 M)	20 (1)	5 (5 M)	21 (1)	NR
Sasaki	2014	Canada	34	16 (10 M)	22 (2)	18 (8 M)	21 (2)	95 (45 d)
Tremblay	2014	Canada	30	15 (15 M)	61 (8)	15 (15 M)	58 (5)	37 (7 y)
Lancaster	2016	USA	52	26 (26 M)	18 (2)	26 (26 M)	18 (2)	24 h
Meier	2016	USA	86	40 (30 M)	20 (1)	46 (30 M)	20 (2)	1, 8, 32 d
Strain	2017	USA	47	25 (25 M)	61 (12)	22 (22 M)	60 (12)	NR
Cubon	2018	USA	53	13 (10 M)	21	40 (34 M)	NR	2 d, 2 w, 2 m
Murdaugh	2018	USA	28	16 (16 M)	16 (1)	12 (12 M)	16 (1)	<7 d
Mustafi	2018	USA	58	30 (30 M)	19 (1)	28 (28 M)	20 (1)	24-48 h
Rubin	2018	USA	98	98 (49 M)	22	NR	NR	<1 y
Jang	2019	USA	76	61 (61 M)	17 (1)	15 (15 M)	17 (1)	NR
MacDonald	2019	USA	46	22 (9 M)	13 (2)	24 (11 M)	13 (2)	1 m, 6 m
Terry	2019	USA	40	20 (20 M)	53 (7)	20 (20 M)	50 (8)	22-47 y
Shapiro	2020	Australia	43	17 (10 M)	13 (2)	26 (21 M)	13 (3)	14 d
Ware	2020	USA	19	10 (10 M)	46 (10)	9 (9 M)	43 (9)	NR
Wu	2020	USA	219	82 (69 M)	19 (1)	137 (109 M)	19 (1)	24-48 h, asymptomatic state (9 d), unrestricted RTP (16 d)
deSouza	2020	USA	71	14 (5 M)	20 (2)	57 (28 M)	19 (1)	45.9 (29.2 m)
Major	2021	Australia	53	26 (26 M)	24 (1)	27 (27 M)	24 (1)	> 6 m
Wright	2021	Australia	30	14 (8 M)	23 (3)	16 (9 M)	24 (3)	24-48 h, 2 weeks
Crasta	2022	USA	25	12 (7 M)	15 (2)	13 (9 M)	16 (2)	2 weeks
Ly	2022	USA	77	29 (15 M)	20 (2)	48 (22 M)	20 (2)	<72 h
Pinky	2022	Canada	65	45 (37 M)	15 (2)	20 (16 M)	15 (2)	8 (3 d)

Abbreviations: d = days; m = months; M = male; NR = not reported; w = weeks; y = years.

**Table 3.** Summary of sports, DTI methodology, DTI results, and clinical measures for the included studies.

First author	Sport	Level of play	DTI quantification	DTI results	Clinical measures
Zhang	Boxing	Professional	ROI	↑ whole-brain diffusivity, ↓ FA in CC and posterior limb of IC	NR
Cubon	Football, hockey, rugby	Collegiate	TBSS	↑ MD in diffuse WM tracts of the left hemisphere, including inferior LF, superior LF, FOF, retrolenticular IC, posterior TR, AR	Injury severity
Hart	Football	Professional	Voxel-based	↓ FA in frontal and parietal regions, CC, L temporal lobe	IQ, Attention and cognition, Language, Visuospatial skills, Depression
Strain	Football	Professional	TBSS, voxel-based, ROI	Depressive symptoms correlate negatively with FA in forceps minor, right frontal aslant tract, right uncinate fasciculus, and L superior LF	Depressive symptom severity (Buckley 3-factor model)
Bazarian	Football	Collegiate	Voxel-based	↓ FA from baseline to postseason with greater changes visible with ↑ peak rotational acceleration	Cognition (ImPACT), Balance (Postural stability)
Sasaki	Ice hockey	Collegiate	TBSS	↑ FA in bilateral CR, posterior limb of the IC, superior frontal WM, and R superior temporal WM; ↓ RD in bilateral posterior limb of the IC, bilateral CR, R CP, R anterior limb of IC, R superior and inferior temporal WM; ↑ AD in L CR	Cognition (ImPACT), SCAT2
Tremblay	Football, ice hockey	Collegiate	TBSS	↓ FA in CC, forceps minor, R inferior LF, and R inferior FOF; ↑ MD in anterior body and genu of the CC and forceps minor; ↑ RD in temporal and occipital aspects of the R inferior LF	Cognition, Verbal fluency, Processing speed, Visual and verbal episodic memory, Depression
Lancaster	Football	High school, collegiate	TBSS	↓ MD in the genu and splenium of the CC, cingulum, IC, superior LF, inferior LF, inferior FOF, CP, R external capsule, R thalamic WM	Injury and Symptoms Severity (SCAT-3, BESS, SAC)
Meier	Various Contact Sports	Collegiate	ROI	↑ FA in R sagittal striatum, bilateral superior CP, R retrolenticular IC, bilateral superior LF, R forceps minor, inferior FOF, L posterior CR; ↓ RD in bilateral superior LF, R forceps minor, and inferior FOF	NR
Strain	Football	Professional	Voxel-based, whole-brain	FA values correlated with BNT performance in the forceps major and minor; voxel-wise correlation between BNT score and FA in clusters along bilateral anterior CR, L posterior CR, and CC	Visual-Confrontation Naming (BNT)
Cubon	Football, ice hockey	Collegiate	TBSS	↑ MD and RD in R superior/inferior LF, R corticospinal tract, R/L inferior FOF, R AR, R/L anterior TR, R/L UF, and forceps major/minor	Anxiety (GAD7), General Cognition (ImPACT), SCAT2
Murdaugh	Football	High school	Group Connectometry	Altered diffusion along a segment of the corticospinal tract and the superior LF in the acute phase of SRC. No differences between the SRC group and control group were seen at follow-up imaging. No correlations with DTI metrics and neurocognitive performance on the ImPACT or TSS	Visual/Verbal memory composites (ImPACT)
Mustafi	Football	Collegiate	TBSS, ROI	↑ MD in genu, body, and splenium of CC, anterior/posterior CR, and superior LF; AD correlated with BSI and SCAT; FA correlated with higher cognitive component of SAC; BSI positive correlation with AD and MD in CC, anterior/superior/posterior CR, superior FOF	SCAT (symptom severity), BSI-soma (physical symptoms), BSI-anxiety, BSI-depression, SAC (cognition), BESS (postural stability), BSI (psychological distress)
Rubin	Soccer	Amateur	Voxel-based, whole brain	↓ FA in genu and splenium of CC and pons; association with FA and heading stronger in women with ↓ FA in L occipital, R parietal, R orbitofrontal WM, L superior LF, R cingulum, R cerebral peduncle; ↑ FA in L temporal WM in men; greater heading exposure had lower WM AD over a greater volume in men than in women	NR
Jang	Football	High school	TBSS	↑ MD and ↓ FA in CC and superior LF	NR
MacDonald	Soccer, Basketball, Martial Arts	Youth recreational	ROI	↓ FA in L middle frontal gyrus WM and L superior parietal gyrus	ImPACT, Verbal/visual memory, Reaction time, Weschler Intelligence Scale for Children, Delis-Kaplan Executive Function System Test-Verbal Fluency Module, Symbol Digit Modalities Test (oral and written processing speed), Grooved Peg Board Test (fine motor speed and dexterity), Health Behavior Inventory (HBI), Patient Health Questionnaire-9 (PHQ-9) for depression, the Generalized Anxiety Disorder-7 (GAD-7), and the Adolescent Sleep-Wake Scale (ASWS)
Terry	Football	High school	TBSS, ROI	↑ MD in the anterior limb of the IC; global FA was positively correlated with immediate memory, language, and cognition	Wechsler Test of Adult Reading (WTAR), Symptom Assessment Scale (SAS), Repeatable Battery for the Assessment of Neuropsychological Status (RBANS), Green‚ Medical Symptom Validity Test (MSVT)
Shapiro	Various	Recreational, high school	TBSS	No difference between the groups (delayed v. normal recovery) in FA, AD, RD, or MD in 11 unique WM tracts	WISC-IV Coding and Digit Span, Contingency Naming Task
Ware	Boxing	Professional and amateur	ROI and Voxel-based	↓ FA across regions of the CC; ↑ MD in the CC genu; no significant difference for RD values across all CC locations; years boxed strongly correlated with ↓ FA and ↑ RD; ↑ FA in CC splenium associated with high anxiety and depression symptoms; ↑ RD related to slow visuomotor tracking and visual scanning; poor fine motor dexterity related to ↓ MD and ↑ FA	Beck Anxiety Inventory (BAI), Center for Epidemiological Studies-Depression (CES-D), Trail Making Test, Fine motor dexterity (Grooved Pegboard)
Wu	Football, soccer, lacrosse	Collegiate	TBSS, ROI	↑ MD in WM at 24-48 h after injury and beyond the point when concussion became asymptomatic; positive correlation with MD elevation and worse outcome on BSI; ↑ RD at acute concussion and not during asymptomatic periods; inverse correlation with MD and time required for concussed athletes to reach asymptomatic state	SCAT4, Balance Error Scoring System (BESS) 18 (Postural stability), BSI (psychological health)
deSouza	Soccer, tennis, basketball, volleyball, baseball, softball, track and field, cross country	Collegiate	ROI	↑ FA in the R hippocampal portion of the cingulum bundle, bilateral external capsule, bilateral fornix/stria terminalis, bilateral UF, bilateral posterior CR, L posterior TR, R superior CR, bilateral superior LF, L sagittal stratum, and bilateral tapetum associated with better TMT B-A, SDMT, and immediate memory; ↓ MD in the bilateral anterior CR, bilateral anterior limb of the IC, genu of the CC, L external capsule, R fornix/stria terminalis, bilateral posterior CR, bilateral posterior limb of the IC, bilateral posterior TR, bilateral retrolenticular IC, bilateral superior CR, bilateral superior LF, bilateral sagittal stratum, and R tapetum associated with better performance on DMT and delayed memory but worse performance on choice RT; ↓ RD in bilateral anterior CR, bilateral anterior limb of the IC, genu, bilateral external capsule, bilateral fornix/stria terminalis, bilateral UF, bilateral posterior CR, L posterior limb of the IC, bilateral posterior TR, bilateral retrolenticular IC, bilateral superior CR, R superior FOF, bilateral superior LF, L sagittal stratum, and bilateral tapetum associated with better performance on DMT and immediate memory, worse performance on choice RT	Symbol Digit Modality Test (SDMT), Trail Making Test Parts A (TMT-A) and B (TMT-B), immediate and delayed memory, simple and choice reaction time (RT), and the Graded Symptom Checklist (GSC)
Major	Australian football	Amateur	TBSS	↓ FA, and ↑ RD, throughout major WM tracts (superior LF, CC, and corticospinal tract); no significant differences in AD	NR
Wright	Australian football	Amateur	TBSS, ROI	↑ FA in genu of CC at 48 h, resolved by 2 w; ↓ ADC at 48 h (corticospinal tract, IC, pars triangularis) that resolved with time, but still some decreases seen at 2 w; ↓ RD at 48 h (genu, body, splenium of CC, external capsule, inferior/superior LF, CR) and RD changes less pronounced at 2 w; M > F symptom severity and WM disruption	Number of symptoms, severity
Crasta	Various, both contact and non-contact	Adolescent recreational or competitive sports	Voxel-based, whole brain	Initial visit had significantly ↑ MD of the superior CR; ↓ FA of CC post-medical clearance compared to initial visit but no difference in MD; no correlation with age and FA and MD measures of the superior CR or CC; no group differences on mean FD or MD in superior CR and CC	PANESS motor function
Ly	Contact club sports	Collegiate	Voxel-based	Male athletes showed ↑ FA than female athletes in the forceps minor, bilateral cingulum, R anterior TR, and R UF	SCAT3
Pinky	Ice hockey	High school	TBSS, voxel-based, ROI	↓ MD in central CC; no significant difference in AD	NR

Abbreviations: AD = axial diffusivity; AR = acoustic radiation; BESS = Balance Error Scoring System; BNT = Boston Naming Test; CC = corpus callosum; CP = cerebellar peduncle; CR = corona radiata; FA = fractional anisotropy; FOF = fronto-occipital fasciculus; IC = internal capsule; ImPACT = Immediate Post-Concussion Assessment and Cognitive Testing; LF = longitudinal fasciculus; L = left; MD = mean diffusivity; NR = not reported; R = right; RD = radial diffusivity; ROI = region of interest; SAC = Standardized Assessment of Concussion; SCAT2 = Sport Concussion Assessment Tool 2; TBSS = tract-based spatial statistics; TMT = Trail Making Test; TR = thalamic radiation; UF = uncinate fasciculus; WTAR = Wechsler Test of Adult Reading; WISC-IV = Wechsler Intelligence Scale for Children—Fourth Edition; d = days; m = months; w = weeks; y = years.

There was notable variation in the time from SRC to DTI scan, which spanned from 24 hours to over 22 years. Several clinical outcome measures were assessed, with the SCAT (6/26 studies) and Immediate Post-Concussion Assessment Testing (ImPACT) (5/26) often used to evaluate symptom severity and cognitive function, respectively. The most frequently reported DTI parameter was FA (20/26), followed by MD (13/26), RD (10/26), and AD (6/26).

MRI parameters were relatively consistent across studies, though certain differences were observed in magnetic field strength, device manufacturer, and head coil channels. MRI post-processing methods were also varied. Most studies used tract-based spatial statistics (TBSS) to quantify DTI metrics (14/26 studies), though other techniques such as voxel-wise and region of interest (ROI)-based analyses were also employed.

### DTI findings and clinical correlates

The anatomical regions analyzed included large WM tracts, such as the corpus callosum (CC), corona radiata (CR), internal capsule (IC), longitudinal fasciculus (LF), and uncinate fasciculus (UF). Findings within groups revealed significant variation in DTI metrics: both increases and decreases in FA, MD, AD, and RD of diffuse WM tracts were observed among athletes post-SRC relative to controls across studies. The acute phase of injury (within 2-3 weeks of injury) was characterized by heterogenous findings with increases and decreases in DTI metrics observed in athletes across studies.^19–29^ In contrast, decreased FA^30–38^ or increased MD^21^^,^^30^^,^^33^^,^^35^^,^^37^^,^^39^^,^^40^ was consistently found in athletes with chronic (1 month to 1 year post-injury) or remote history of SRC (>1 year post-injury).

Quantifiable metrics on the magnitude of DTI alterations were noted in several studies. In an assessment of acute SRC, Lancaster et al observed 34,807 voxels with significantly decreased MD (*P* < .05) in 26 concussed high school and collegiate athletes relative to controls at 24 hours post-injury.^19^ These voxels were predominantly localized to regions of the corticospinal tract (CST) and superior LF. In contrast, Cubon et al identified 12 348 total voxels with significantly higher RD and MD values (*P* < .02) in diffuse anterior-posterior WM regions of 13 varsity college athletes at 2 days post-injury relative to their baseline scans.^21^ These voxels spanned areas of the superior/inferior LF, inferior fronto-occipital fasciculus (FOF), CST, acoustic radiation, anterior thalamic radiations, UF, and forceps major/minor, with comparable magnitude changes between anterior and posterior regions within each subject. Wu et al found 4834 and 1936 WM voxels with significantly elevated MD and RD (*P* < .05) in 82 concussed athletes relative to controls at 24-48 hours post-injury.^25^ MD and RD were elevated by 5.6% and 6.7% in these voxels corresponding to regions of the CC. Similarly, Mustafi et al observed 3397 voxels localized to the CC, CR, and superior LF with a 5%-7% increase in MD (*P* < .05) among 30 American football players compared to controls within 48 hours of SRC.^23^

In the chronic phase of SRC, FA reductions and MD elevations are consistently observed. Zhang et al identified significantly reduced FA in the splenium of CC of 49 boxers relative to controls (0.84 ± 0.04 vs 0.87 ± 0.04, *P* < .05), as well as in the posterior limb of IC (0.71 ± 0.04 vs 0.75 ± 0.06, *P* < .05).^30^ Ware et al also demonstrated reduced FA in the splenium of CC of 10 boxers relative to controls (0.56 ± 0.01 vs 0.58 ± 0.02, *P* = .003), as well as increased MD in the genu of CC (0.82 ± 0.03 vs 0.80 ± 0.01, *P* = .038).^37^ Cubon et al found a large cluster (961 voxels) with increased MD corresponding to regions of the left inferior LF, inferior FOF, retrolenticular IC, posterior thalamic radiation, acoustic radiation, and superior LF in 10 varsity college athletes relative to controls at least 1 month post-SRC (*P* < .05).^39^ Terry et al reported higher MD in 10 voxels localized to the right anterior limb of IC of 40 former high school football players compared to controls over 22 years after retirement from the sport (*t* = 4.18, *P* = .033).^40^

Alterations in DTI metrics were associated with clinical measures that evaluated symptom severity and time of recovery. Elevations in FA were related to improved cognitive function (ρ = 0.85, *P* < .05), greater performance on neuropsychological tests (*r* = 0.58, *P* > .02), and better executive function (ρ = 0.85, *P* < .05).^23^^,^^41^^,^^42^ High MD was linked to deficits in motor function (*r* = 0.43, *P* = .034) and symptom severity (ρ = 0.83, *P* < .05).^23^^,^^27^ Low RD was correlated with worse choice reaction time (bootstrap ratio > 2, *P* < .05) and high AD was positively associated with symptom severity (ρ > 0.83, *P* < .05).^23^^,^^42^ Notably, negative findings on the relationship between DTI parameters and SRC clinical outcome measures were also reported.^22^^,^^43^

Five studies utilized DTI to investigate whether changes in WM integrity are related to depressive symptoms, a possible sequela of SRC.^31^^,^^36^^,^^37^^,^^41^^,^^44^ In a study of 26 retired National Football League (NFL) athletes with a history of SRC, Strain et al reported negative correlations between FA of numerous ROIs and symptom severity as assessed by the Beck Depression Inventory II (*r* < −0.550, *P* < .01).^44^ Notably, FA of the forceps minor was able to differentiate depressed from non-depressed athletes with 100% sensitivity and 95% specificity. Another study from the same group identified significant FA reductions in bilateral frontal and parietal regions of 14 retired NFL athletes with cognitive or mood impairments relative to asymptomatic athletes (*P* < .05).^31^ Mac Donald et al observed decreased FA in the left middle frontal gyrus of 22 youth athletes with persistent depressive symptoms despite cognitive improvement 6 months post-SRC (*P* < .001).^36^ In contrast, Ware et al reported an association between FA in the splenium of the CC and depressive symptoms in 10 boxers (*r* = 0.78, *P* = .022).^37^

Three studies investigated sex-related differences in DTI metrics associated with SRC.^26^^,^^28^^,^^34^ Ly et al demonstrated higher FA in diffuse WM tracts of males compared to females in a study of 29 concussed university athletes at 72 hours post-SRC.^28^ Wright et al found significantly greater WM disruption among males relative to females at 48 hours and 2 weeks post-SRC in a study of 14 athletes recruited from amateur Australian football clubs (*P* < .001).^26^ In a study of 98 amateur soccer players, Rubin et al identified more regions with reduced FA associated with heading exposure in females relative to males (8 vs 3 regions, >100 contiguous voxels, *P* < .01).^34^ However, no significant sex-related differences in associations between heading and either RD or AD were found.

### Repetitive concussion and longitudinal studies

The effect of repetitive sports-related head trauma on microstructural alterations was evaluated in 5 studies.^32^^,^^33^^,^^35^^,^^37^^,^^40^ Tremblay et al reported widespread WM differences in 15 retired athletes with a history of multiple SRCs compared to controls, particularly decreases in FA as well as increases in MD and RD of fronto-parietal networks (eg, superior LF).^33^ Ware et al demonstrated associations between boxing history and DTI metrics in a study of 10 amateur/professional boxers.^37^ Factors such as the number of professional bouts, years of sparring, and number of professional knockouts with and without loss of consciousness correlated with reduced FA, increased MD, and increased RD in subregions of the CC (*r* > |0.4|, *P* < .05).

Bazarian et al conducted a prospective observational study of 10 college football players who underwent DTI at pre-season, postseason, and after 6 months of no-contact rest.^32^ The authors found a positive correlation between the number of voxels with reduced FA and number of sub-concussive head impacts, and this association persisted even after 6 months of no-contact rest (*r* = 0.91, *P* < .001 for head hits with a peak rotational acceleration exceeding 4500 rad/sec^2^). A similar retrospective study by Jang et al demonstrated greater WM microstructural disruptions among high school football athletes relative to controls and showed that these markers of injury (eg, reduced FA) correlated with cumulative exposure to head acceleration events (*r* = 0.231, *P* < .05).^35^ In contrast, Terry et al found only minimal differences in DTI metrics among 20 former high school football players with a history of 2 or more SRCs relative to non-concussed controls.^40^

Longitudinal studies revealed time-dependent alterations in DTI metrics following SRC. Meier et al found acute FA increases in diffuse WM tracts, particularly the superior LF, of 40 collegiate athletes that persisted at 1.6, 8.3, and 32.1 days post-injury (Cohen’s d ≥ 0.67, *P* ≤ .01 at all time points).^20^ Murdaugh et al also identified increased FA in the superior LF (FD*r* = 0.20) and CST (FD*r* = 0.27) of 16 male high school football players at acute injury, but these alterations resolved 21 days later.^22^ Similarly, Pinky et al showed that acute elevations in FA and reductions in MD in the CC of 29 youth ice hockey players with SRC were not evident at 41 days post-injury (*P* < .038).^29^

Chronic microstructural impairment was also observed in a longitudinal study by Wu et al, who found persistently elevated MD in regions of the CC of 82 collegiate athletes at 24-48 hours, asymptomatic state (∼9 days), 7 days after return-to-play, and 6 months post-injury.^25^ MD was highest at acute concussion (5% relative to controls) and decreased from the initial time point to asymptomatic state, plateauing thereafter (*P* < .05). The number of voxels with significant MD also dropped from 4834 at the initial time point to 1763 at 6 months post-SRC. A study of 13 college athletes who sustained a SRC by Cubon et al demonstrated elevations in MD and RD at 2 days, 2 weeks, and 2 months post-injury (*P* < .02).^21^ The affected regions consisted of anterior and posterior WM regions spanning both hemispheres, and the pattern of recovery followed a posterior-to-anterior trend.

### Novel analytical methods

Four studies performed single-subject analysis to evaluate individual alterations in DTI metrics.^20^^,^^21^^,^^32^^,^^35^ In contrast to inter-group comparisons, this approach accounts for potential inter-individual variability in DTI findings post-SRC. For example, while Bazarian et al identified persistent WM changes in athletes following exposure to repetitive head injuries at the group level relative to controls, individual DTI findings revealed that some athletes returned to their baseline levels.^32^ Another study also assessed subject-specific DTI alterations in comparison to baseline (ie, pre-injury) scans, thus enabling a clear delineation of microstructural injury due to SRC.^21^

One study by Ly et al investigated the integration of DTI with existing clinical methods to improve the diagnostic accuracy of SRC.^28^ The authors developed a classifier that combined cognitive measures with DTI parameters, particularly MD, attaining an overall accuracy of 74% and sensitivity of 64% for the detection of acute concussion in 29 university athletes. While self-reported symptom assessment remains a more accurate diagnostic method based on existing criteria, the findings present an objective improvement in diagnostic performance over either neuroimaging or cognitive evaluation alone.

### Quality assessment

**Figure 2.**
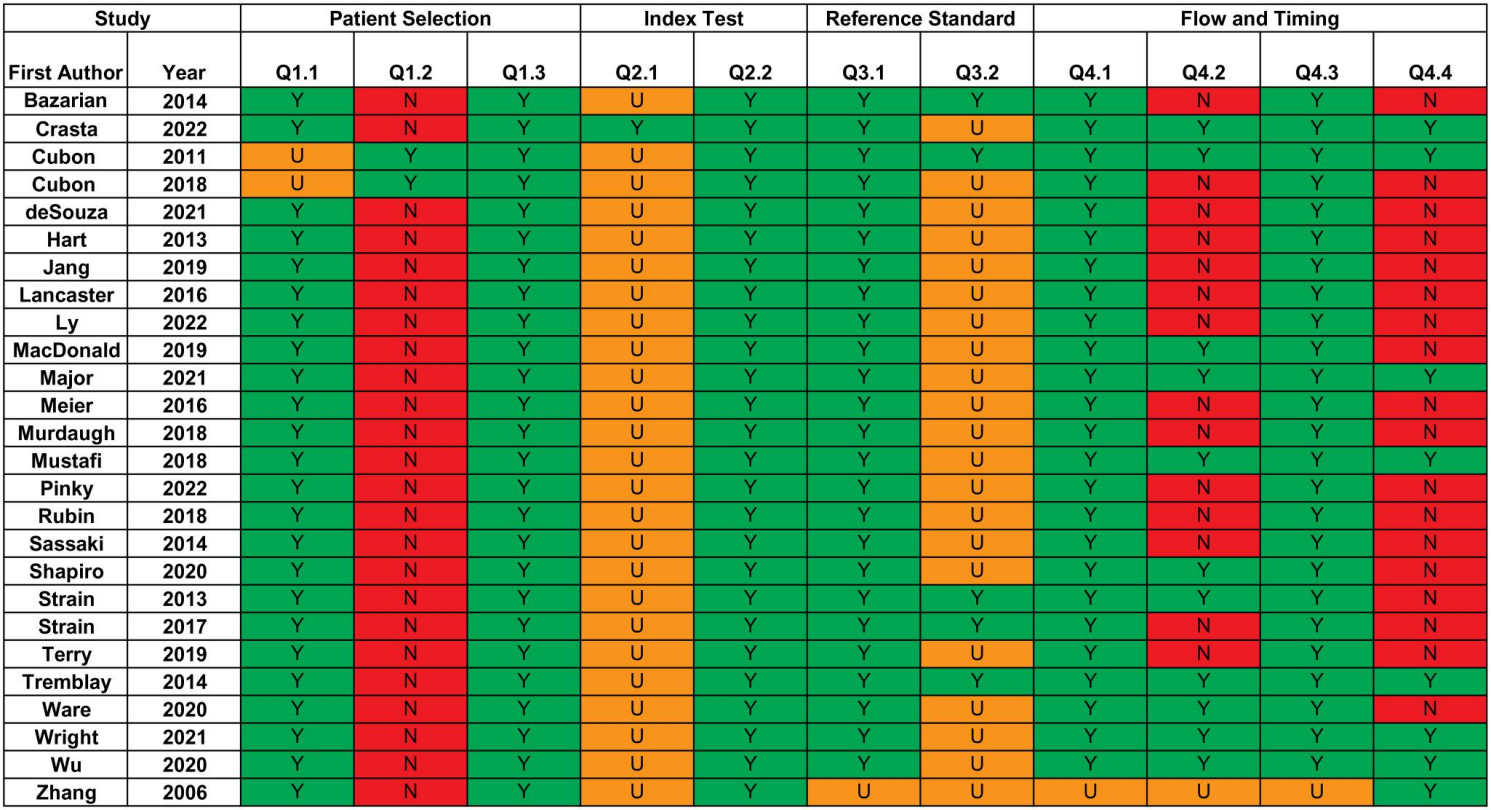
Summary of quality assessment of risk of bias and clinical applicability using the QUADAS-2 protocol.

Most studies were assigned a low risk of bias in all 4 QUADAS-2 domains (patient selection: 100.0%; index test: 100.0%; reference standard: 19.2%; flow and timing: 26.9%). The reference standard domain demonstrated the greatest risk of bias due to a lack of reported information regarding whether clinical outcome measure(s) were interpreted without knowledge of the DTI scan. As an absence of such a separation could theoretically influence the analysis of results, many studies were assigned an “unclear” risk. The flow and timing domain also showed concern for bias as studies used an inconsistent time interval between injury, clinical assessment, and DTI scan. Moreover, studies with serial imaging often lost subjects to follow-up. Overall, quality assessment of the included studies showed a satisfactory risk of bias across domains. The summary of the quality assessment is presented in Figure 2.

## Discussion

This systematic review synthesizes current evidence regarding DTI applications in SRC, analyzing findings from 26 original research studies. The reviewed literature demonstrates evidence of microstructural alterations following SRC, with DTI metrics showing heterogenous acute and consistent chronic WM changes. Notably, these microstructural alterations often persist beyond the resolution of clinical symptoms and “return-to-play” clearance, potentially correlating with adverse long-term neurological outcomes. While the compiled evidence supports DTI's utility as a sensitive biomarker of SRC, significant methodological heterogeneity across studies necessitates further standardization and validation. This variability in study design and outcomes underscores the need for more rigorous investigation to establish DTI’s role in concussion management.

Post-SRC alterations in DTI parameters reflect distinct pathophysiological processes affecting neuronal architecture and function. In the acute phase, cytotoxic edema induces both intracellular and extracellular compartmental swelling, modifying water molecule movement along axonal pathways. These changes manifest as measurable alterations in anisotropy and diffusivity metrics.^45^ The magnitude and direction of DTI parameter changes depend specifically on the intracellular-to-extracellular water ratio, which varies according to the severity of shear strain forces sustained during injury.^46^ In chronic phases, progressive Wallerian degeneration characteristically presents as reduced FA values accompanied by elevated MD measurements, consistent with axonal deterioration and myelin loss.^47^^,^^48^ While patterns of Wallerian degeneration can also be seen on conventional MRI in the chronic setting, DTI’s ability to detect microstructural changes in acute and chronic injury may provide additional diagnostic and prognostic value. This temporal evolution of DTI findings aligns with our review’s observations, which demonstrate variable acute phase alterations but more consistent patterns in chronic injury stages.

Methodological heterogeneity across studies represents a significant source of variability in DTI findings. Demographic variations in study populations—including age stratification (youth to retired athletes), gender distribution, sport type, and competition level—contribute to distinct patterns of microstructural alteration following head trauma.^49^^,^^50^ For instance, non-pathological WM alterations at the global and tract-specific level have been associated with both age and gender in cognitively healthy adults.^51^ Children and adults demonstrate physiological differences in patterns of anisotropy and diffusivity.^52^

The cumulative effect of repetitive concussive impacts, particularly evident in professional and retired athletes, may also alter WM microstructural integrity as assessed by DTI.^33^^,^^37^ Repetitive concussion can influence DTI metrics in both short-term (eg, after one season) and long-term (eg, retired athletes) settings.^32^^,^^33^ Sport-specific biomechanical profiles further differentiate injury patterns; boxing-related impacts, for example, generate predominantly rotational rather than translational head acceleration vectors compared to football-related concussions.^53^ Additionally, certain sports carry an elevated risk of concurrent cervical injuries, such as whiplash syndrome, which can compound microstructural alterations detectable by DTI.^54^

Technical variability in MRI acquisition parameters and DTI analysis protocols introduces another layer of methodological inconsistency that may account for divergent findings across studies. For instance, Hunter et al obtained discrepant results between ROI and voxel-based methods on the magnitude and direction of FA alterations in the CC of TBI patients.^55^ The authors concluded that an ROI-based method is superior for detecting the full extent of microstructural alterations in the CC, as the magnitude of damage revealed by ROI was greater than that demonstrated by the voxel-based method (*z* = −3.15 vs *z* = −1.41). In a DTI study of 49 children, Kumpulainen et al reported that decreasing the number of diffusion-encoding directions reduced both accuracy and precision of all DTI scalar values.^56^ A minimum of 18 diffusion directions was recommended to achieve reliable DTI results with TBSS.

A critical methodological limitation pervading SRC research is the insufficient documentation of individual subject characteristics, particularly regarding concussion history and injury severity metrics.^57^ Within our reviewed literature, assessment of prior concussions predominantly relied on self-reported data, introducing potential recall bias and measurement inconsistency. Only a subset of studies implemented objective head impact monitoring systems, such as helmet-mounted accelerometers, to quantify exposure parameters.^32^^,^^35^ This may help explain why certain correlations between DTI metrics and head exposure were observed in studies that used objective measures, but not in other studies that relied on subjective patient recall (in some cases >22 years since playing sport) to define repeated SRC.^40^

The significant variability in clinical outcome measures used across studies presents an additional limitation to the qualitative assessment performed in this systematic review. Different parameters were widely assessed such as the ImPACT, SCAT, and the Brief Symptom Inventory (BSI) scores. This restricts the feasibility of using conventional SRC clinical outcome measures as a reference standard and warrants the need for future investigations to implement consistent parameters. Moreover, the inconsistent definition of a control group (eg, healthy athletes or non-athletes) may affect the quality of results obtained from the included studies.

The emergence of single-subject DTI analytical approaches presents a promising methodology for capturing inter-individual variability that traditional group-level analyses may obscure.^21^ Implementation of these individualized assessment protocols within prospective longitudinal study designs represents a crucial step toward validating DTI's clinical utility in personalized concussion evaluation and recovery monitoring. For instance, a direct comparison of a patient’s baseline DTI scan with a scan at 24 hours, 1 month, or 1 year post-SRC could provide clinicians a quantifiable marker of an athlete’s degree of injury and ability to return-to-play.

## Conclusion

The collective evidence from this systematic review highlights DTI's value as a promising biomarker for both acute microstructural injury and chronic WM disruption, substantiating its potential in SRC evaluation. DTI's capacity to detect persistent neural alterations in the absence of clinical symptoms offers unprecedented opportunities for identifying at-risk populations and monitoring recovery trajectories.^19^^,^^20^^,^^25^ The observed microstructural changes and their topology in DTI maps provide valuable insights into the underlying mechanisms of post-concussive functional, cognitive, and psychiatric sequelae. However, substantial methodological heterogeneity across studies—encompassing variations in design, analytical approaches, clinical assessment scales, and outcome measures—presents significant challenges for data interpretation and clinical implementation. Future research initiatives must prioritize standardization of MRI protocols using state-of-the-art hardware and post-processing algorithms, implementation of longitudinal designs, creation and curation of publicly available databases of normal and sport-related TBI subjects encompassing repetitive TBI cases (eg, TRACK-TBI), and validation of individualized analytical approaches to definitively establish DTI’s role in clinical concussion management. Such methodological refinements will be essential for translating DTI's promising technological capabilities into meaningful improvements in SRC diagnosis, monitoring, and outcome prediction.

## Supplementary Material

tzaf024_Supplementary_Data

## Transparency, rigor, and reproducibility summary

The study design and analytic plan was not formally pre-registered, but the team member with primary responsibility for this analysis certifies that the plan was pre-specified. A total of 397 articles were obtained from 3 databases: PubMed, Web of Science, and Scopus. 166 duplicates were removed, and an additional 159 studies were eliminated from analysis based on title and abstract screening. Of the remaining 67 full-text articles, 41 studies were excluded due to ineligibility based on our inclusion and exclusion criteria. The criteria we used are based on established standards in the literature on DTI and SRC. Overall, 26 studies met all criteria for our final qualitative synthesis. For each study, established DTI measures and relevant clinical outcome measures were collected. Shiv Patil had full access to all the data in this study and takes responsibility of the data and the accuracy of data analysis. The authors agree to provide the full content of the manuscript on request by contacting the authors of this study.
