# Supplementary material for: Clinical utility of diffusion tensor imaging in sport-related concussion: a systematic review
Source: BJR Open. 2025 Oct 8;7(1):tzaf024. doi: 10.1093/bjro/tzaf024 (PMC12538676; doi:10.1093/bjro/tzaf024)
Supplement: tzaf024_Supplementary_Data [file tzaf024_supplementary_data.zip › Supplementary File 1 Search Strategies.docx]

**Search Strategies for PubMed, Web of Science (WoS), and Scopus**

PubMed: ("Brain Concussion"[MeSH Terms] OR "concuss*"[tiab]) AND ("sport"[tiab] OR "Sports"[MeSH Terms] OR "Athletes"[MeSH Terms] OR "athlete*"[tiab]) AND ("Diffusion Tensor Imaging"[MeSH Terms] OR "DTI"[tiab] OR "Diffusion tensor imag*"[tiab] OR "diffusion imag*"[tiab] OR "tractography"[tiab] OR "diffusion tensor MRI"[tiab]) AND ("Diagnosis"[MeSH Terms] OR diagnos*[tiab])

WoS: (TS=”concuss*”) AND ((TS=”sport”) OR (TS=”athlete*”)) AND ((TS=”diffusion tensor imag*”) OR (TS=”DTI”) OR (TS=”diffusion imag*”) OR (TS=”tractography”) OR (TS=”diffusion tensor MRI”)) AND (TS=”diagnos*”)

Scopus: ((TITLE-ABS-KEY("concuss*")) AND (((TITLE-ABS-KEY("sport")) OR (TITLE-ABS-KEY("athlete*"))) AND (((TITLE-ABS-KEY("diffusion tensor imag*")) OR (TITLE-ABS-KEY("tractography")) OR (TITLE-ABS-KEY("DTI")) OR (TITLE-ABS-KEY("diffusion tensor MRI"))) AND (TITLE-ABS-KEY("diagnos*")))
