# Supplementary material for: Clinical utility of diffusion tensor imaging in sport-related concussion: a systematic review
Source: BJR Open. 2025 Oct 8;7(1):tzaf024. doi: 10.1093/bjro/tzaf024 (PMC12538676; doi:10.1093/bjro/tzaf024)
Supplement: tzaf024_Supplementary_Data [file tzaf024_supplementary_data.zip › Supplementary File 3 QUADAS-2 Guide.docx]

**QUADAS-2 Guide of Questions and Judgement Criteria**

**QUADAS-2 Questions**

**Q1.1** Was a consecutive or random sample of patients enrolled?

**Q1.2** Was a case-control design avoided?

**Q1.3** Did the study avoid inappropriate exclusions?

**Q2.1** Were the DTI results interpreted without knowledge of the results of the SRC clinical

outcome measures?

**Q2.2** If a threshold was used for the DTI scan, was it pre-specified?

**Q3.1** Is/are the SRC clinical outcome measure(s) likely to correctly classify SRC?

**Q3.2** Was/were the SRC clinical outcome measure(s) interpreted without knowledge of the results of the DTI scan?

**Q4.1** Was there an appropriate interval between the DTI scan and the SRC clinical outcome measure(s)?

**Q4.2** Did all patients complete the SRC clinical outcome measure(s)?

**Q4.3** Did all patients complete the same SRC clinical outcome measure(s)?

**Q4.4** Were all patients included in the final analysis?

**Criteria for Judgments**

We defined the index test as the DTI scan and the reference standard as the SRC clinical outcome measure(s). Our choice to use these measures as a reference standard was informed by the following factors: (1) other conventional neuroimaging modalities lack the sensitivity to accurately diagnose SRC, which limits their utility as comparators for DTI; and (2) the studies included in this review specifically aimed to assess DTI in relation to SRC clinical outcome measures (13).

For each section, risk of bias was assessed consistent with the Cochrane Handbook for Systematic Reviews of Interventions: all ‘Yes’ responses to signalling questions prompted assignment of a ‘LOW’ risk of bias, all ‘No’ responses prompted assignment of a ‘HIGH’ risk of bias, and all ‘Unclear’ responses prompted assignment of an ‘UNCLEAR’ risk of bias (20).

Cases of mixed ‘Yes’, ‘No’, and ‘Unclear’ responses were handled in the following manner:

1. Patient Selection: A ‘LOW’ risk of bias was assigned when two answers were ‘Yes.’ A ‘HIGH’ risk of bias was assigned if two answers were ‘No.’ If both answers were ‘Unclear,’ an ‘UNCLEAR’ risk of bias was assigned. In cases where the answers included a mix of ‘Yes,’ ‘No,’ and ‘Unclear,’ the risk of bias was deemed ‘UNCLEAR.’
2. Index Test: A combination of ‘Yes/No’ or ‘No/Yes’ was assigned an ‘UNCLEAR’ risk of bias. Combinations such as ‘Unclear/Yes,’ ‘Yes/Unclear,’ ‘Unclear/No,’ or ‘No/Unclear’ were classified as a ‘LOW’ risk of bias, as ‘Unclear’ responses typically reflect the study author’s omission of complete details regarding the DTI scan, rather than indicating a significant risk of bias.
3. Reference Standard: Combinations of ‘Unclear/Yes,’ ‘Yes/Unclear,’ ‘Unclear/No,’ or ‘No/Unclear’ were assigned an ‘UNCLEAR’ risk of bias due to mixed evidence. A ‘No/Yes’ combination was assigned a ‘HIGH’ risk of bias, as question 3.1 is critical for bias assessment in this review; if the reference standard (SRC clinical outcome measure(s)) was inaccurate, it would compromise the comparison with DTI outcomes and thus undermine the review's objectives. A ‘Yes/No’ combination would also result in an ‘UNCLEAR’ risk of bias.
4. Flow and Timing: A combination of two ‘No’ answers and two ‘Yes’ answers were assigned an ‘UNCLEAR’ risk of bias due to the mixed evidence. If there were three ‘Yes’ answers, this would indicate a ‘LOW’ risk, while three ‘No’ answers would result in a ‘HIGH’ risk. Similarly, three ‘Unclear’ answers would be classified as an ‘UNCLEAR’ risk. Two ‘Unclear’ answers were also assigned an ‘UNCLEAR’ risk of bias.
